# The Metabolic Mechanisms of Breast Cancer Metastasis

**DOI:** 10.3389/fonc.2020.602416

**Published:** 2021-01-07

**Authors:** Lingling Wang, Shizhen Zhang, Xiaochen Wang

**Affiliations:** ^1^ Department of Breast Surgery, Zhejiang Provincial People’s Hospital, Hangzhou, China; ^2^ Department of Surgical Oncology and Cancer Institute, Second Affiliated Hospital, Zhejiang University School of Medicine, Hangzhou, China; ^3^ Institute of Translational Medicine, Zhejiang University School of Medicine, Hangzhou, China

**Keywords:** breast cancer, metabolism, metastasis, molecular mechanisms, metabolic phenotypes, glycolysis, hypoxia

## Abstract

Breast cancer is one of the most common malignancy among women worldwide. Metastasis is mainly responsible for treatment failure and is the cause of most breast cancer deaths. The role of metabolism in the progression and metastasis of breast cancer is gradually being emphasized. However, the regulatory mechanisms that conduce to cancer metastasis by metabolic reprogramming in breast cancer have not been expounded. Breast cancer cells exhibit different metabolic phenotypes depending on their molecular subtypes and metastatic sites. Both intrinsic factors, such as *MYC* amplification, *PIK3CA*, and *TP53* mutations, and extrinsic factors, such as hypoxia, oxidative stress, and acidosis, contribute to different metabolic reprogramming phenotypes in metastatic breast cancers. Understanding the metabolic mechanisms underlying breast cancer metastasis will provide important clues to develop novel therapeutic approaches for treatment of metastatic breast cancer.

## Introduction

Breast cancer is the most common malignant tumor and the second capital reason for cancer death among women worldwide (1, 2). Metastatic breast cancer, not the primary tumor, is responsible for more than 90% cancer-related deaths (3). A SEER based study showed that for metastatic breast cancer patients: 30–60% have metastases in the bone, 21–32% in the lung, 15–32% in the liver and 4–10% in the brain. Moreover, the preferred metastatic sites appear to depend on the specific pathological subtypes of primary breast cancers (4).

Recently, increasing evidence point out that cancer is not only a genetic disease but also a metabolic disease, in which oncogenic signaling pathways participate in energy regulation and anabolism to support rapidly spreading tumors (5). In this sense, metabolic reprogramming is considered a hallmark of cancer (6, 7). Notably, metabolic reprogramming and its complex regulatory networks also affect the tumorigenesis and progression of breast cancer (8). Considered as a high heterogeneous disease, breast cancer includes four main intrinsic molecular subtypes: Luminal A, luminal B, HER2-positive, and triple-negative breast cancer (TNBC). Each subtype has different proliferation and metastasis capabilities, as well as metabolic genotypes and phenotypes (9–16) (**Table 1**). Specifically, TNBC cells possess particular metabolic traits characterized by high glycolysis and low mitochondrial respiration (22). HER2-positive tumors display higher glutamine metabolic activity and higher lipid metabolism than other subtypes (15, 20). Nevertheless, metabolic changes may not only be varied in different breast cancer subtypes, but also diverged relying on the interplay of cancer cells with the complex microenvironment (16, 23).

**Table 1:** Metabolic differences in different breast cancer subtypes.

	Expression level	Luminal A subtype	Luminal B subtype	HER2+ subtype	Basal-like/TNBC
Glucose metabolism					
	G6PD and 6PGL (17)	lower		Higher	
	6PGDH (17)				only
	HIF-1α, IGF-1, and MIF (18)			Notedly increased	
	GLUT-1 and CAIX (18)				Notedly Increased
Amino acid metabolism					
	Stromal GLS1 (19)	Lowest		Highest	
	Stromal GDH (19)	Lowest		Highest	
	Tumoral GDH (19)			Highest	lowest
	Tumoral ASCT2 (19)	Lowest		Highest	
	Stromal PSPH and SHMT1 (14).	Lowest		Highest	
	Stromal and tumoral GLDC (14)			Highest	lowest
Lipid metabolism					
	Tumoral PLIN1, CPT-1A, and FASN (20)			Highest	lowest
	Tumoral FABP4, and ACOX-1 (20)			Highest	
		ER+ tumor		ER- tumor	
		Inhibition of 27-hydroxycholesterol synthesis decreases cell proliferation in ER+ cancers but not in ER- cancers (12).		Higher ACAT activity, higher caveolin-1 protein levels, greater LDL uptake, and lower *de novo* cholesterol synthesis (10); Products of *de novo* fatty acid synthesis, such as palmitate-containing phosphatidylcholine, were high (11).	
Genes related with metabolism		Luminal B tumors displayed higher glutamine metabolic activity driven by *MYC* than Luminal A tumors (15).		Highest glutamine metabolic activity and higher *MYC* amplification (15).	Loss of *p53* collaborates with MYC_high_/TXNIP_low_-driven metabolic dysregulation to drive the aggressive clinical behavior in TNBC but not in other subclasses of breast cancer (21).

ER, estrogen receptor; TNBC, triple-negative breast cancer; G6PD, glucose-6-phosphate dehydrogenase; 6PGL, 6-phosphogluconolactonase; GLS1, glutaminase 1; GDH, glutamate dehydrogenase; ASCT2, alanine-serine-cysteine transporter2; HIF-1α, hypoxia‐inducible factor 1α; SHMT1, serine hydroxymethyltransferase 1; IGF-1,insulin-like growth factor-1; MIF, macrophage migration inhibitory factor; GLDC, glycine decarboxylase; PLIN1, perilipin-1; FASN, fatty acid synthase; CPT-1A, carnitine palmitoyltransferase-1; FABP4, fatty acid binding protein 4; ACOX-1, acyl-CoA oxidase 1; GLUT-1 glucose transporter protein-1; CAIX, carbonic Anhydrase IX; ACAT, acetyl-CoA acetryltransferase; sPLA2, secreted phospholipase A2; TXNIP, thioredoxin-interacting protein.

This review addresses the current knowledge on the crosstalk between metabolic reprogramming and metastatic process in breast cancer. A better understanding of the metabolic mechanisms driving breast cancer metastasis may provide clues for discovering new anticancer therapeutics.

## Overview of Metabolic Programming in Breast Cancer

### Glucose Metabolism

**Figure 1:**
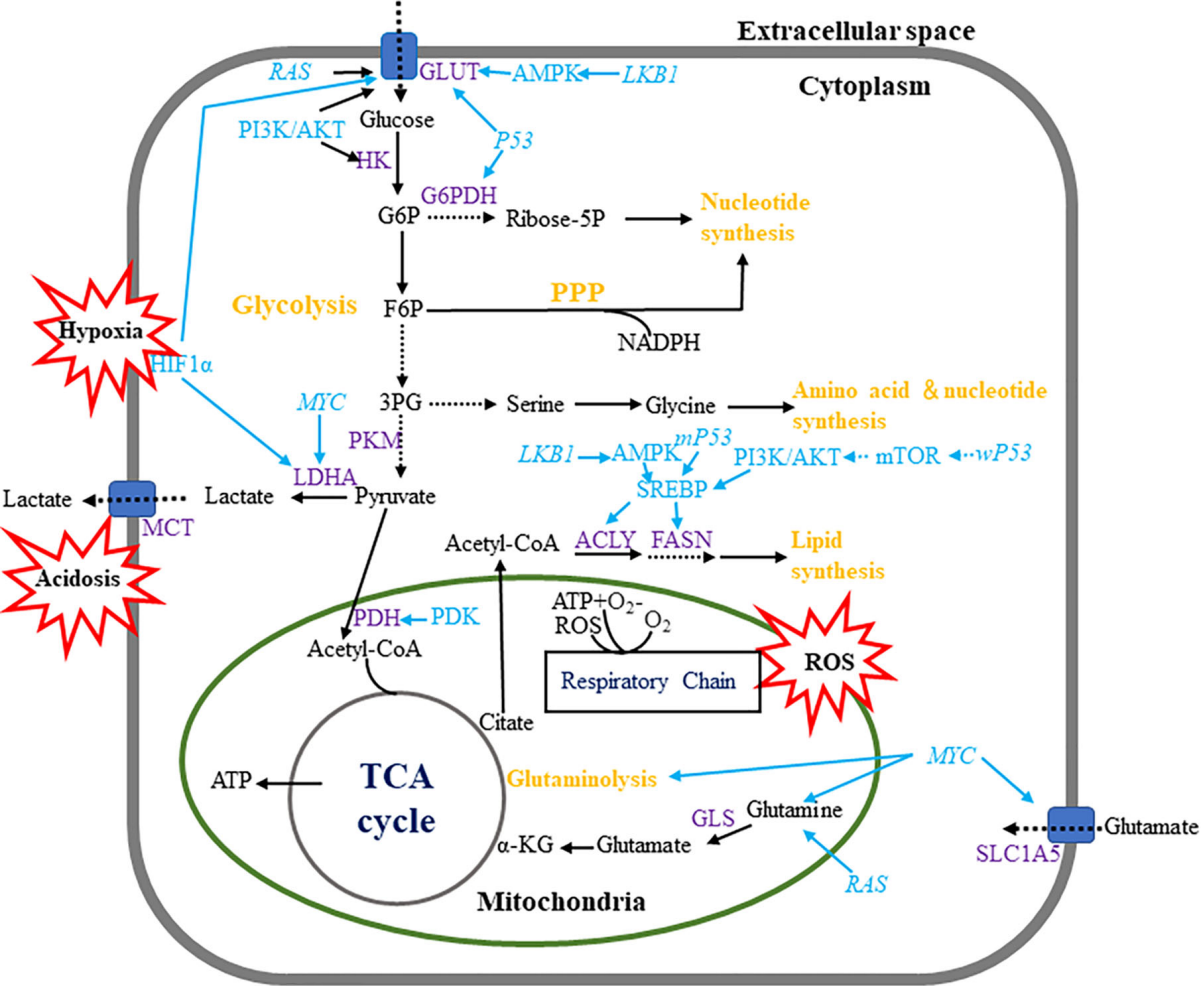
Metabolic pathways in breast cancer cells. Breast cancer cells enhance metabolism of glucose, amino acid lipid by regulating multiple metabolic pathways. Breast tumor cells mainly use aerobic glycolysis to produce ATP and utilize the pentose phosphate pathway to produce macromolecules such as NADPH. Wild-type and mutant *P53* have contrary effects in monitoring fatty acid metabolism. Hypoxia, acidosis and ROS are regarded as important events which influence multiple metabolic pathways.

In response to external growth signals, normal cells in a rapidly proliferating state activate assorted signaling pathways to suppress oxidative phosphorylation (OXPHOS), and advance glycolysis and anabolic metabolism for cell growth. Cancer cells is able to hijack this mechanism to meet developmental needs even if there are no external signals (24) (**Figure 1**). Different from normal cells where glycolysis and OXPHOS are always negatively correlated, cancer cells possess these two modes coexisting to disparate degrees (25). Moreover, unlike normal cells, which mainly produce adenosine triphosphate (ATP) from glucose-derived pyruvate by OXPHOS through the TCA cycle, most cancer cells depend on glycolysis to generate energy even under aerobic conditions (26). It was found that tumors displayed dual metabolic natures that tumor cells could switch from the aerobic glycolysis back to OXPHOS phenotype upon lactic acidosis (27). Furthermore, some tumors exhibit two-compartment tumor metabolism, called the reverse Warburg effect or metabolic coupling, which indicates that glycolytic metabolism in the cancer-related stroma sustains the adjacent cancer cells. Such metabolic phenotype will contribute to chemotherapy resistance, and also explain the contradictory phenomenon of high mitochondrial respiration and low glycolysis rate in some tumor cells (28–30). Moreover, a large sample data study showed luminal subtype correlated with reverse-Warburg/null phenotypes that are metabolically inactive, while TNBC correlated with Warburg/mixed phenotypes that are metabolically active (31). Additionally, hypoxic environment in breast tumors brings about increased production of reactive oxygen species (ROS) (32), at the same time, induced hypoxia-inducible factor 1 (HIF-1) is able to boost glucose metabolism to maintain the redox homeostasis (33).

Glucose transport cross cell membrane through the glucose transporter proteins (GLUTs) and different GLUTs expression in breast cancers are related to dissimilar pathological grades and prognosis. GLUT1-5 and GLUT12 are functionally in breast cancer cells (34–37), and GLUT1 appears to play the most important role (38). Interestingly, TNBC had the highest expression of GLUT1 when contrasted with other subtypes, suggesting the highly active metabolic status in TNBC (18). Moreover, some critical glycolysis-related enzymes, such as hexokinase (HK) and lactate dehydrogenase-A (LDHA), are highly activated in breast cancer and related to cancer growth and progression (39, 40).

The pentose phosphate pathway (PPP) is another way of oxidative decomposition of glucose besides glycolysis and TCA cycle, which produces nicotinamide adenine dinucleotide phosphate (NADPH), ribose phosphate, fructose-6-phosphate (F6P) to make cancer cells satisfy their anabolic needs and respond to oxidative stress (41). Proteins involved in PPP are distinctively expressed in different molecular subtypes of breast cancers. For example, the expression of glucose-6-phosphate dehydrogenase (G6PD) and 6-phosphogluconolactonase (6PGL) were elevated, implying a more activated PPP in HER2 subtype than other subtypes of breast cancer (17). It has been suggested that the expression of G6PD and transketolase (TKT) are positively correlated to the decreased overall and relapse-free survival in breast cancer (42).

### Amino Acid Metabolism

Glutamine and its metabolic intermediates such as antioxidants nicotinamide adenine dinucleotide (NADH), and glutathione (GSH), participate in energy supply, supplement glucose metabolism and help cells resist oxidative stress to uphold proliferation and progression of tumor cells (43, 44). Some cancer cells exhibit “glutamine addiction” that cannot survive in the absence of exogenous glutamine (45). More importantly, certain oncogenic transcription factors, such as *c-MYC* and *RAS*, can increase the cancer cell glutamine metabolic activity by upregulating glutamine transporters such as alanine-serine-cysteine transporter 2 (ASCT2) and enzymes participating in the conversion of glutamine-to-glutamate, such as glutaminase (GLS)-1 (46). For example, *c-MYC* activates the expression of ASCT2 and GLS-1 under the induction of lactic acid, leading to elevated glutamine uptake and catabolism in cancer cells (47). Notably, a metabolomic analysis indicted that breast tumor tissues had a higher glutamate‐to-glutamine ratio (GGR) than normal tissues, especially in estrogen receptor (ER) negative tumors and the GGR levels dramatically correlated with ER status and tumor grade (48). The glutamine metabolism-related proteins, such as GLS-1, glutamate dehydrogenase (GDH), and ASCT2 were found to be highly expressed in HER2-positive breast cancer than other subtypes, which indicated that HER2-positive breast cancer had the highest glutamine metabolism activity (19).

One-carbon metabolism, also known as network of folate utilization reactions, participates in multiple metabolic pathways such as amino acid biosynthesis and degradation, *de novo* nucleotide biosynthesis, and methylation and reductive metabolism (49). It has been widely accepted that one-carbon metabolism acts a pivotal part in supporting the high proliferative rate of tumor cells (50). Folate (vitamin B9), a carrier of one-carbon units, and other B vitamins, such as B6 and B12, take part widely in one-carbon metabolism, which is requisite for DNA biosynthesis and methylation (51). Although the relationship between folic acid intake and the risk of breast cancer is still controversial, a recent meta-analysis, analyzing 23 prospective studies, found that an increment of folate intake decreased the risk of ER-, ER-/PR-, premenopausal breast cancer and had the preventive effects against breast cancer in individuals with alcohol consumption (52).

In addition to glutamine, upregulation of serine/glycine metabolism closely connected with folate metabolism is relevant to high proliferation of tumor cells and poor prognosis of patients (50). Tryptophan and arginine are involved in the manipulation of immunity and tolerance, which are generally deregulated in cancers (53). The activity of arginase, the key enzyme catalyzing L-arginine, in breast tumor environments is strengthened, which generates an unfavorable milieu for T cell adaptability (54).

### Lipid Metabolism

The fatty acids (FAs) and lipid metabolic programming also play significant parts in promoting breast cancer growth and progression (55). Cancer cells maintain a highly proliferative state by activating the uptake of exogenous lipids and lipoproteins, or by reinforcing *de novo* lipid and cholesterol biosynthesis, showing active lipid and cholesterol metabolisms (56). Moreover, tumor cells mostly rely on *de novo* fatty acid synthesis (FAS) to satisfy the augmented demand for membrane metabolism in favor of rapid growth and proliferation. The expression of fatty acid synthase (FASN), a key enzyme essential for the FAS, is elevated in breast cancer (57), and its upregulation appears to be connected with cancer development, recurrence and poor prognosis (58), suggesting the augmented FAS activity is important for breast cancer progression. Notably, FASN was found to be expressed highest in HER2-positive breast cancer and lowest in TNBC at both cell and tissue levels (20, 59). It has been assumed that a two-way regulatory system between FASN and HER2, the “HER2-FASN axis”, may enhance breast cancer proliferation, metastasis and chemoresistance (60). Sterol regulatory element-binding protein (SREBP)-1, a lipogenic transcription factor, can regulate FASN expression by binding with the FASN promoter site (61, 62). And phosphatidylinositol-3-kinase (PI3K)/AKT/mammalian target of rapamycin (mTOR) and mitogen-activated protein kinase (MAPK) signal transduction pathways are also likely to regulate FASN expression (63, 64). Under hypoxic conditions, *FASN *gene is upregulated due to the arousal of AKT and SREBP-1 in breast tumor cells (65). Inhibition of MAPK pathway and mTOR inhibitor rapamycin both can decrease FASN expression in breast cancer cells (66, 67).

## Overview of Metastasis in Breast Cancer

Tumor metastasis is a sequential multi-step process, which includes local invasion, intravasation, migration through the lymphatics or blood vessels, extravasation and colonization giving rise to the formation of metastases in distant organs (68). Particularly, organ-specific colonization hinge on the dynamic and mutual interrelation between tumor cells and tumor microenvironment (TME), comprised by varieties of non-cancerous cells such as immune cells, endothelial cells, fibroblasts, adipocytes, together with extracellular matrix (ECM) and soluble factors (69). In addition to the linear metastasis model, breast cancers prefer to the parallel metastasis model, which means that breast cancer cells begin to spread in the early stages of tumor development (70), and the spread of cancer cells may be independent of the progression of the primary tumors (71). Studies have shown that the genetic changes of the bone marrow disseminated breast cancer cells are usually not identical to their corresponding primary tumors (72). Different breast cancer subtypes have been found to show different metastatic sites preference governed by different molecular mechanisms (73). The molecular characteristics of breast cancers and target tissues appear to confirm the organotropism of metastasis (74). All the breast cancer subtypes are apt to develop bone metastasis, luminal A subtype is regarded as a risk factor for recurrence in the bone (75), and luminal B subtype is more likely to have bone as a first relapse site when compared to other subtypes (76). Moreover, the incidence of luminal subtype tumors to have bone metastasis is much higher (80.5%) than HER2-positive tumors (55.6%) and basal-like tumors (41.7%) (77). While luminal B and basal-like subtypes present higher levels of lung-specific metastasis (78). Compared with the HER2-negative subtype, the HER2-positive subtype is more often observed with liver metastases (4). Another study showed that basal-like tumors had a higher rate of metastasis to the brain, lungs and distant lymph nodes, while the rate of liver and bone metastasis is much lower (79).

### The Process of Metastasis

The step one of the metastasis is that tumor cells break away from the tumor bed and migrate from the stroma into the bloodstream (80). In order to leave the primary tumor and invade surrounding tissues, these tumor cells need to reduce their tight cell adhesion through undergoing epithelial-mesenchymal transitions (EMT) (81, 82). EMT is typified by loss of epithelial traits (including cell polarity and cell-cell junctions) and acquisition of mesenchymal traits (including fibroblastic spindle-shaped morphology) to increase the mobility of tumor cells. EMT also links to cancer metastasis with stem cell properties (83, 84). Moreover, the integrin-mediated adhesion and debonding interactions with matrix components is critical for regional migration. And the intratumoral blood vessels characterized by increased permeability allow cancer cells to enter the systemic circulation readily (85).

After escaping from the original tumor site to blood circulation, breast tumor cells begin to migrate to remote organs. The first obstacle encountered by circulating tumor cells is the blood vessel wall, especially endothelial cells. In some organs, such as bone marrow and liver, microvessels are composed of sinuses with strong permeability, which make cancer cells easier to break through (86). Whereas in most other organs, including brain, endothelial cells form a continuous barrier that prevents cancer cells from penetrating freely. Platelets and white blood cells can help tumor cells pass through the vasculature by forming complexes with tumor cells through L- or P-selectin (87, 88). As such, increased expression of selectin ligands by tumor cells is well connected with metastatic progression and bad prognosis (89). The induction of angiopoietin-like 4 (ANGPTL4) by transforming growth factor-beta (TGFβ)/small mother against decapentaplegic (SMAD) signaling pathway in cancer cells is reported to enhance their subsequent retention in the lungs and empower breast cancer cells to destroy lung capillary wall and form pulmonary metastases (90). Chemokines in target cell tissues can also induce directed cell migration, initiate signal pathways, and monitor cytoskeletal rearrangment and adhesion (91).

Adjusting to new environment is another hurdle for circulating tumor cells (CTCs) to form metastasis. Disseminated cancer cells will spring up in targeted tissues and organs through a way that is significantly different from their origins. Cancer cells must acquire new capabilities, especially the ability to interact with cells in the ECM and new microenvironment. Tumor cells form a two-way connection with circumferent stroma in the early stage of invasion and after that, tumor-stroma interaction helps the tumor to develop toward metastasis (6).

### Pre-Metastatic Niche

An appropriate microenvironment, namely, pre-metastatic niche, can be established in secondary tissues and organs before metastases occurring through a complicated mechanism by interaction between the primary tumors and organs stromal components (92). Kaplan et al. emphasized the role of tumor-mobilized bone marrow-derived cells (BMDCs) in developing a satisfactory microenvironment for lung metastatic colonization. The factors, such as vascular endothelial growth factor (VEGF), and placental growth factor (PlGF), released by the primary tumor act on the bone marrow mesenchymal stem cells to induce the BMDCs to reach the expected metastasis site before the disseminated tumor cells arrive (93). Hiratsuka et al. demonstrated that matrix metalloproteinase (MMP)-9 is particularly motivated in premetastatic lung endothelial cells and macrophages mediated by primary tumors *via* the VEGFR-1/Flt-1 pathway, which is important for lung metastasis (94). The integrin β1/α5/JNK/c-JUN signaling pathway in cancer cells is able to upregulate the higher matrix stiffness-induced lysyl oxidase like (LOXL)-2, then subsequently promote production of fibronectin, expression of MMP-9 and C-X-C motif chemokine ligand (CXCL)-12 and recruitment of BMDCs to encourage pre-metastatic niche establishment (95). Chemokines binding to specific receptors on the target cell membrane help to recruit immune cells into the tumor microenvironment, thereby managing immune surveillance, angiogenesis, invasion and metastasis (96). The CXCL-12/C-X-C motif chemokine receptor (CXCR)-4 axis provides a fit microenvironment before breast cancer bone metastasis formation (97). Carmen et al. suggested that HIF-1 is a crucial regulatory factor inducing breast cancer metastatic niche forming through activation of several elements of the lysyl oxidase (LOX) family, which catalyze collagen cross-linking in the lungs before BMDC recruitment (98). Dickkopf (DKK)-1 suppresses prostaglandin endoperoxide synthase (PTGS)-2-induced macrophage and neutrophil recruitment to lung metastases by antagonizing cancer cell non-classical WNT/Planar cell polarity (PCP)-RAC1-JNK signaling, whereas it encourages breast-to-bone metastasis by modulating classical WNT signaling of osteoblasts (99).

### Organotropism

The site-specific metastasis of breast cancer is related to subtypes and divergent gene signatures of metastatic cancer cells. Functional studies have identified many key genes that mediate breast cancer organ-specific metastasis, and the expression of these genes in the primary tumor is likely to forecast the patient’s organ-specific metastasis (100, 101).

Bone is the most frequent site of breast cancer metastasis (73). Bone metastasis is usually connected with osteolytic-type lesions as a result of the overactive bone resorption mediated by osteoclasts (102). Integrin complexes, such as integrin αvβ3, α4β1 and α5, play important roles in the attraction and adhesion of breast tumor cells to the bone (103–105). Some clinical, genetic, and functional evidence suggest that the SMAD tumor suppressor pathway may diverted into potent pro-metastatic factor in breast cancer, and signaling through the SMAD pathway can facilitate breast cancer bone metastasis (106). Moreover, both hypoxia (via HIF-1α) and TGFβ signaling can independently stimulate the VEGF and CXCR4 expression to drive breast cancer bone metastases (107). In basal-like TN breast cancer, CCL20 promotes bone metastasis by raising the secretion of MMP-2/9 and increasing the receptor activator of nuclear factors-kappa B (NF-κB) ligand/osteoprotegerin ratio in breast cancer and osteoblastic cells (108).

The second most common metastatic site of breast cancer is the brain (73). Brain metastasis of breast cancer can be located in the parenchymal brain (around four-fifths) or in leptomeningeal region (109). CTCs need to break through the blood-brain barrier (BBB), interplay with the local microenvironment to survive, and then set up brain metastatic colonies. CD44, VEGF and CXCR4 can impair endothelial integrity to raise the transendothelial migration of tumor cells (110). Angiopoietin-2 (Ang-2) expression is elevated in brain microvascular endothelial cells (BMECs) and secreted Ang-2 can increase BBB permeability by disrupting tight junction protein structures between ZO-1 and Claudin-5 in TNBC models of brain metastasis (111). Cyclooxygenase (COX)-2, heparin-binding epidermal growth factor-like growth factor (HBEGF), and ST6GALNAC5 are all able to help tumor cell pass through the BBB (112). Additionally, astrocytes and microglia are related with brain metastases. Astrocytes-derived factors, such as MMP-2 and MMP-9, are able to enhance the migration and invasion of breast tumor cells, thus leading to brain metastasis (113). Similarly, microglia can also be stimulated by culturing with cancer cells, so that it boosts cancer cell colonization in a WNT-dependent manner (114).

Compared to other metastatic lesions, lung metastasis generally show phenotypes of aggressive growth and invasiveness (101). EGFR, COX2, MMP-1, and MMP-2 expressed in breast cancers jointly facilitate lung metastasis by promoting the angiogenesis, emancipating cancer cells into the circulation and breaking through lung capillaries (115). Studies have determined that compared with primary breast cancer, the degree of pyruvate carboxylase (PC)-dependent anaplerosis in lung metastasis of breast cancer is higher, as a result of responding to the lung microenvironment (116). Bone morphogenetic proteins (BMPs) secreted by lung resident cells can restrict cancer development by turning cancer cells into a dormant state, while Coco and GALNTs derived from lung metastatic breast tumor cells are able to inhibit the effect of BMPs and reactivate dormant tumor cells to seed in the lung, thereby leading to metastasis (117).

Breast cancer cells preferred to liver-specific homing display unique transcriptional profiling (118). The status of ER, progesterone receptor (PR) and HER2 between the primary and liver metastatic tumors of breast cancer can be changed after treatment (119). Development of breast cancer liver metastasis is reported to be associated with the activation of β-catenin-independent WNT signaling (120). A model for breast cancer liver metastasis was established involving diverse factors from breast tumor cells and the liver microenvironment such as integrin complexes, HIFs and LOX (121).

### Breast Cancer Stem Cells and Dormant Cells

Breast cancer stem cells (BCSCs), a small number of cells with self-renewal and unlimited replication capabilities, have been shown in numerous cancer models to be involved in tumor development and metastatic dissemination. Moreover, the occurrence of BCSCs with the properties of stemness, EMT and drug resistibility, is the main cause for cancer recurrence and treatment failure (122). Multiple researches revealed that several signaling pathways, such as WNT/β-catenin and Notch, contribute significantly to the development of BCSCs (123). Devon A et al. showed that early metastatic breast cancer cells had unique stem-like gene expression characteristics and prefer to proliferate and differentiate to produce advanced metastatic disease at the single-cell level (124). Additionally, BCSCs isolated from primary human breast cancers possess the advanced metastasis potential and the CD70+ subpopulations appear to preferentially mediate lung-specific metastasis by enhancing self-renewal potential (125).

After colonizing the distant metastatic site, BCSC can enter into a metastatic dormant state, showing the halted proliferation and activated cellular stress response, while maintaining metabolic activity (126–128). The dormant phenotype is able to be reversed by manipulating of intrinsic and/or extrinsic factors and then the proliferative program restarts *in vivo* (129, 130). However, the biological mechanisms of cell dormancy and re-awakening are still elusive (131). The dormant state is regarded as a high risk of cancer recurrence and is supposedly limit the efficiency of chemotherapy. Targeting the metastatic dormancy, therefore, could be an promising treatment strategy to improve long-term control of cancer progression (132).

## Metabolic Reprogramming and Organ-Specific Metastasis

Metabolic plasticity is one of the important characteristics that distinguishes the tumor cells with high metastatic potentiality from non-metastatic tumor cells. Metastatic cancer cells always operate multiple metabolic pathways concurrently, thus they can adjust the application of diverse pathways according to their adaptive requirements (133, 134). Cancer cells are challenged by diverse environmental and cellular stresses during metastatic progression (135). Strikingly, cancer cells are capable of manipulating one or more metabolic pathways according to their stage in the metastatic cascade and the site they metastasize (133, 136–139). For instance, extracellular acidification by the release of CO_2_, lactic acid and other organic acids from metabolically vigorous tumor cells promotes intravasation of cells from the primary tumor. Once tumor cells enter the circulatory system, they produce NADPH and GSH through the PPP pathway to protect themselves from oxidative stress. The coordination of the metabolism between cancer cells and adjacent microenvironment is critical for successful colonization of distant sites and survival during dormancy. Most importantly, anabolic metabolism is reactivated in cancer cells to facilitate the growth of macro-metastatic tumors (140). Tumor metabolism reprogram also occurs when tumors progress in order to adapt to lack of sufficient blood supply. While during adaptation to environmental stress, such as cyclic hypoxia, tumor metabolism reprogram contributes to selection of drug-resistant and metastatic clones (141, 142).

**Figure 2:**
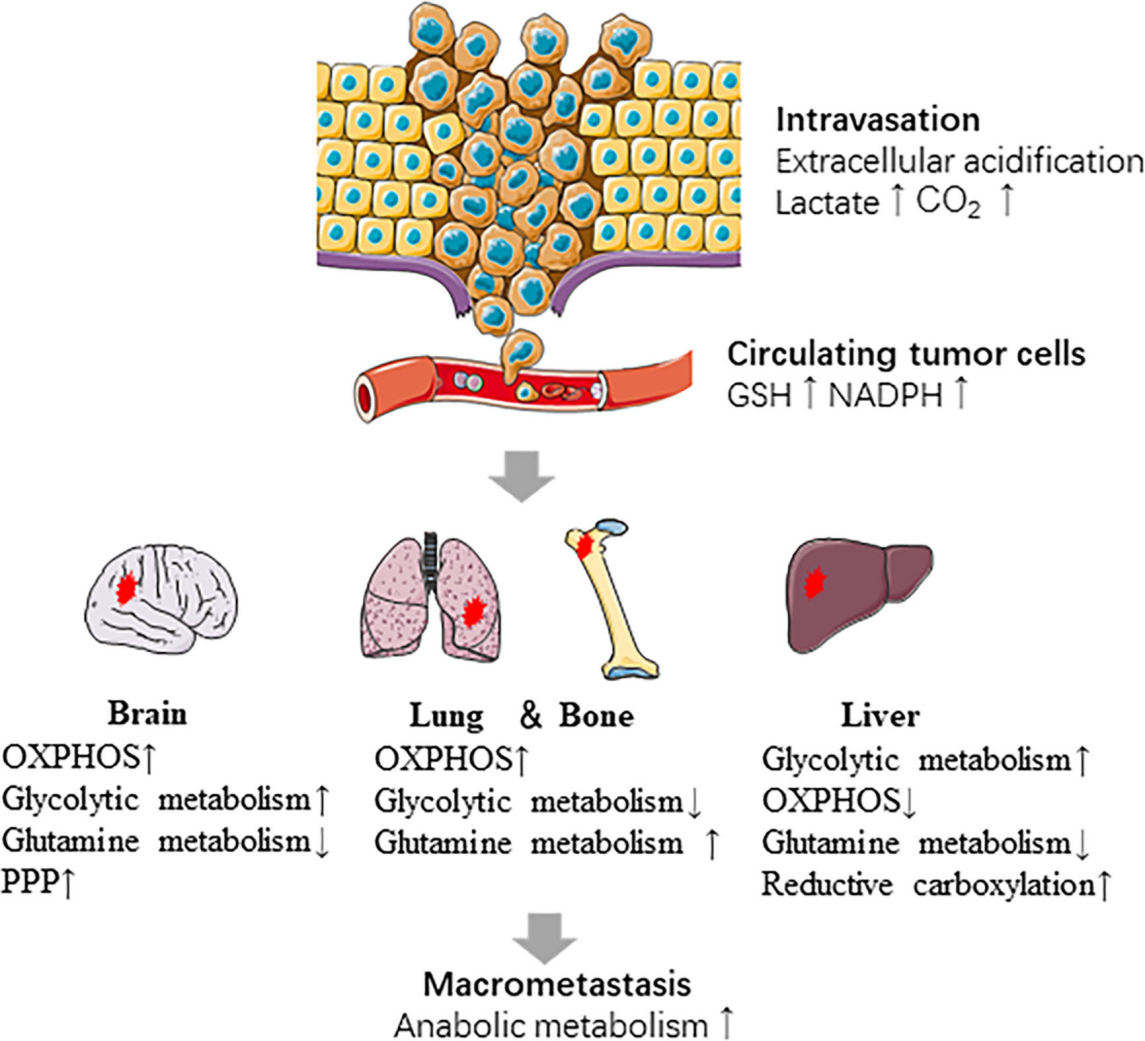
Metabolic reprogramming in the metastatic cascade. Metabolic reprogramming occurs at several steps of metastasis. The intravasation of cells from the primary tumor is promoted by extracellular acidification. CTCs survive in oxidative stress by producing NADPH and GSH. Cancer cells show different metabolic characteristics based on the sites which they metastasize. Last, anabolic metabolism is reactivated during macro-metastatic tumor proliferation.

Primary breast tumor cells exhibit metabolic heterogeneity and participate in different metabolic reprogramming according to metastatic sites (**Figure 2**). Liver-metastatic breast cancer cells display a distinct metabolic reprogramming characterized by accumulation of glucose-derived lactate and reduction in the TCA cycle and OXPHOS (138). In brain metastatic breast cancer, the significant metabolic changes are mainly the enhanced glycolysis, mitochondrial respiration and the PPP. Intriguingly, breast cancer cells metastasized to the brain are less sensitive to glucose deficiency (136), which may attribute to upregulation of glutamine and branched chain amino acid oxidation (143).

Preference for metastatic sites is determined by many factors, including proximity to the primary tumor site and breast cancer subtype. Not only pro-metastatic genes in these subtypes, but also related metabolic mechanisms are closely related to the propensity of metastatic organs. Monica et al. utilized Raman spectroscopy (RS) and Multivariate Curve Resolution-Alternating Least Squares (MCR-ALS) analysis to study biochemical differences between metastasis tropisms in two TNBC cell lines and showed that bone metastasis tropism was characterized by the increase of amino acids and the decrease of mitochondrial signal, while high lipid and mitochondrial (cytochrome C and RNA) levels for lung metastasis (144). NETosis is an important neutrophil function that can promote liver metastasis of breast cancer and different pro-metastatic neutrophil populations are highly metabolically adaptable, which facilitates the formation of liver metastases (145). The products of pathologically deposited lipids can promote metastasis and nonalcoholic fatty liver disease (NAFLD) activates tumor-induced triglyceride lipolysis in juxtaposed hepatocytes, thereby promoting breast-to-liver metastasis (146). In addition to genetic tendency, metastatic cells that inhabit the brain are adaptive to crosstalk with many different brain residential cells (112, 147). The important role of Notch signaling in breast cancer brain metastasis has been recognized, and it has recently been considered to regulate metabolism (148, 149). Reactive astrocytes promote the metastatic growth of breast cancer stem-like cells by activating Notch signals in the brain and astrocyte-derived cytokines contribute to the metastatic brain specificity of breast cancer cells (150, 151). In addition to the similarity of certain metabolic signaling pathways such as the Wnt/β-catenin pathway, Heregulin-HER3-HER2 signaling and the EGFR/PI3K/Akt pathway, brain metastatic cancer cells also share certain metabolic characteristics with neuronal cells (152). Metastatic cells with neuron-like properties thrive in the brain microenvironment. For example, neurons typically catabolize gamma-aminobutyric acid (GABA) to create NADH to support biosynthetic processes and breast tumor cells with a GABAergic phenotype have a strong growth advantage in the brain by converting GABA to succinate to boost the citric acid cycle (153). Enzymes involved in lipid metabolism may also be the appropriate target to prevent the formation of brain metastases, because oncogenic lipid signaling can promote the metastasis of breast tumor cells to the brain by supporting cell survival, migration, and invasion (154).

## Crosstalk Between Metabolic Regulators and Metastatic Pathways

### Intrinsic Factors: Tumor-related Genes that Regulate Metabolic Pathways in Breast Cancer Metastatic Cascade

#### TP53

*TP53* mutations are very common in breast cancers, especially in triple-negative and HER2-positive subtypes (155). TP53 is recognized to mediate its tumor-suppressive functions by adjusting the expression of genes that promote cell cycle arrest, apoptosis, and senescence (156). Moreover, TP53 is able to suppress tumorigenesis by regulation of metabolism and reactive oxygen species (ROS) production (157). There are several mechanisms involved in TP53-mediated metabolic changes (158, 159). For example, wild-type *TP53* is able to inhibit glycolysis by suppressing the expression of GLUT1, GLUT3, and GLUT4 (160, 161), and regulating the expression of enzymes involved in the glycolytic pathway, such as HK2 (162), phosphofructokinase 1 (PFK1) (163), phosphoglycerate mutase (PGM) (163), pyruvate dehydrogenase (PDH), parkin 2 (PARK2) (164), and pyruvate dehydrogenase kinase (PDK2) (165). *TP53* also regulates mitochondrial respiration in cancer progression. *TP53* loss results in downregulation of mitochondrial respiration and oxidative metabolism, which contribute to the Warburg effect in tumor cells, thus linking to tumor progression (166). Besides, by upregulating the cytochrome c oxidase 2 (SCO2) (167), *TP53* initiates several transcriptional programs to promote the expression of genes related to mitochondrial biogenesis (168), such as apoptosis-inducing factor (AIF) (169, 170) and ferredoxin reductase (FDXR) (171). The expression of GLS-2 is also positively regulated by wild-type *TP53*, as such the conversion of glutamine-to-glutamate increases, which is requisite for supplement of NADPH and GSH (172, 173). In contrast, mutant *TP53* has been proved to drive the glycolysis by activating the RhoA/ROCK/GLUT1 signaling cascade (164), repress the catabolic activities, such as fatty acid oxidation (FAO), by inhibiting 5′-AMP-activated protein kinase (AMPK) pathways, and enhance the anabolic processes, such as enhanced fatty acid synthesis (174).

Wild-type and mutant *TP53* have contrary effects in managing the fatty acid metabolism. Wild-type *TP53* hampers the shunt of the glucose carbon to anabolic pathways by binding to and inhibiting the G6PD, whereas mutant *TP53* is unable to affect the G6PD activity (175). Moreover, wild-type *TP53* appears to negatively control the mTOR pathway and the PPP, therefore governing fatty acid synthesis (175, 176). However, mutant *TP53* enhances lipid synthesis through interacting with SREBPs (177). In particular, *TP53* mutation connects with raised expression of genes involved in mevalonate pathway in human breast cancer, and most importantly, mutant *TP53* upregulates these genes and activates the mevalonate pathway, which is indispensable to keep the malignant status of breast cancer (178).

#### c-MYC

Amplification of *c-MYC* and activation of its downstream effectors are related with high metastatic ability, endocrine resistance and poor disease outcome in breast tumors (179). The c-MYC pathway is well known to enhance the cancer cell growth and proliferation. Its role in the orchestration of metabolic pathways, which provides nutrients and other essential factors to motivate DNA replication and cell division, was recently identified. Specifically, *MYC* amplification mediates the glutamine-related metabolic rewiring in breast cancers, that promotes the excessive uptake of glutamine by inducing the expression of glutamine transporters and glutamine-metabolizing enzymes (180). Such *MYC* amplification-mediated molecular mechanism is specifically upregulated in the luminal B, HER2-positive, and TN breast cancers (15). Moreover, c-MYC activation links to TCA cycle overactivation in HER2-positive and TN breast tumors by increasing the uptake of serine, glycine, and tryptophan and the synthesis of one-carbon units  (181).

Beside, c-MYC and other transcription factors, such as mTOR and HIF-1, can act synergistically to improve glycolysis and promote cancer proliferation (182, 183). c-*MYC* is also a direct target and a coregulator of ERα  (184), it can act synergistically with ERα to induce breast cancer cell proliferation (185). Furthermore, ER regulates the glutamine metabolism by crosstalking with HER2 signaling in a way dependent on c-MYC in aromatase inhibitor-resistant breast cancer cells (186). Other studies have reported that c-MYC drives glucose metabolism in TNBC by inhibiting thioredoxin-interacting protein (TXNIP)—an inhibitor of glycolysis (21).

#### PI3K/AKT/mTOR Pathway

PI3K/AKT/mTOR pathway is an intracellular signaling pathway significant for cell cycle and metabolism involved in cancer progression (187). The activation of PI3K/Akt/mTOR pathway is able to enhance expression of genes related to glucose uptake and glycolysis through normoxic upregulation of HIF-1α (188–191). Activation of mTORC1 is also likely to be a latent mechanism driving the Warburg effect by upregulation of *c-MYC* (182, 183). Moreover, the PI3K/AKT/mTOR pathway can facilitate the expression of lipogenic genes in an SREBP-dependent manner (192), and mTORC1 has been regarded as a vital effector in advancing the trafficking or processing of SREBP to stimulate *de novo* lipogenesis (193). Activation of mTORC1 is adequate to provoke the expression of genes encoding the enzymes of both the oxidative and non-oxidative branches of the PPP, thus activating specific bioenergetic and anabolic cellular processes (194). The PI3K/AKT/mTOR pathway was recently showed to reduce oxidative stress and promote cell survival of breast epithelial cells segregated from the ECM by strengthening flux through the oxidative PPP (195)

*PIK3CA* mutation, which leads to increased PI3K activity, is the most common somatic mutation in breast cancer, and 36% of patients with HR+/HER2- breast cancer are *PIK3CA* mutated (196). It was suggested that crosstalk between the ER and PI3K/AKT/mTOR signaling pathway exists during breast cancer development (197). Estrogens stimulate PI3K/AKT/mTOR pathway to conduct the migratory and invasive features of ER tumors (198, 199). Reciprocally, mTOR signaling monitors the expression and activity of ERα (200). A recent study reported that PI3K pathway repression triggered the activity of the histone-lysine N-methyltransferase 2D (KMTD2), which leads to the activation of ER in breast cancer cells (201). Interestingly, reactivation of AKT/mTOR signaling by using small molecule PI3K antagonists activates the transport of energy-active mitochondria to the cortical cytoskeleton of cancer cells, therefore heightening tumor cell invasion (202). Although PI3K pathway inhibitors reduce cancer growth, they could accidentally increase tumor invasion by inducing reprograming of mitochondrial trafficking, OXPHOS, and promoting cell motility (203). Moreover, suppression of the mTOR-p70S6K axis is able to induce possessing of unique metabolic features, distinguished by high glucose uptake, incremental lactate production, and low mitochondrial respiration, in TNBC cells (22).

#### Estrogen Receptor

More than two-thirds of the breast cancer cases present as ERα-positive, and cancers with ERα-positive without HER2-positive is termed as luminal breast cancer (204). Luminal breast cancer appears to have a metabolic phenotype that balances the glycolysis and OXPHOS, while TNBC is more relying on OXPHOS (22). ER-positive tumors have lower levels of glycine, lactate, and glutamate (high glutamine) and lower GGR with lower levels of glutaminolysis, which suggest that ER is implicated in regulation of tumor metabolism (205). ER plays a central role in metabolic regulation through crosstalk with multiple pivotal regulators and pathways, such as TP53, c-MYC, HIF, Ras/Raf/MAPK and PI3K/AKT/mTOR pathway, enabling tumors to reprogram their metabolism to fit various kinds of environment (16). 17β-estradiol (E2) is capable of increasing the expression of insulin receptors and decreasing the lipogenic activity of lipoprotein lipase in adipose tissue by activating ERα (206). Moreover, E2 and ERα can regulate the metabolism reprogram based on glucose availability. In high glucose conditions, E2 enhances glycolysis *via* enhanced AKT kinase activity and suppresses TCA cycle activity, while in low extracellular glucose conditions, E2 stimulates the TCA cycle *via* the upregulation of PDH activity and suppresses glycolysis to satisfy the energy requirements of the tumor cell (207). Besides, a study employing the nuclear magnetic resonance spectroscopy illustrated that E2 appeared to induce glycolysis, whereas tamoxifen reduced it (208–210). Mechanically, E2 is able to transcriptionally upregulate GLUT1, thus promote glycolysis (210).

Contrary to ERα, ERβ is expressed in more than 50% of normal mammary epithelial cells, but less than 10% of tumor cells in invasive ductal carcinoma (211). In general, expression of ERβ is downregulated or lost in high grade breast tumors, but its relation to clinical outcome does not reach an agreed conclusion (212). In glucose metabolism, ERβ, similar to ERα, seems to enhance glycolysis while repress OXPHOS (213). Most importantly, ERβ is suggested to play a key role in regulating the metabolism of BCSCs, given several glycolysis-related pathways are upregulated in ERβ-activated mammospheres (214).

#### HER2

HER2-positive breast tumors generally exhibit a glycolytic phenotype (215, 216) and display the increased uptake levels of glutamine, glycine, creatinine, and succinate while a reduction in alanine levels (205). Moreover, the expression of FASN, carnitine perilipin-1 (PLIN1), and palmitoyltransferase-1A (CPT1A) are elevated in HER2-positive breast cancers (20). HER2 is involved in multiple signaling pathways that promote glucose utilization (216), regulate LDHA (40) and 6-phosphofructo-2-kinase (PFKFB3) expression levels (217), and induce lactate accumulation in tumors (218). Additionally, HER2 can be translocated to the mitochondria by the intercourse with mitochondrial heat shock protein-70 (mtHSP70), which negatively controls oxygen consumption and thus enhancing glycolysis (219). Inhibition of HER2 pathways by a dual novel EGFR/HER2 inhibitor, KU004, significantly inhibits the Warburg effect by downregulating HK2, thus decreasing cancer cell proliferation (220). Overactivated HER2 signaling results in increased HIF-1α and VEGF expression, which in turn activate the downstream kinase FKBP-rapamycin-associated protein (FRAP), therefore contributing to tumor progression by mediating angiogenesis and metabolic adaptation (221).

#### Breast Cancer Type 1 Susceptibility

*BRCA1*-mutated breast tumors are usually phenotyped as aggressive, high-grade, aneuploidy tumors (222, 223), and with a worse prognosis (224). Loss of BRCA1 function caused by *BRCA1* mutation results in the production of hydrogen peroxide in both epithelial breast tumor cells and adjacent stromal fibroblasts, which is able to promote the onset of a reactive glycolytic stroma, suggesting the metabolic phenotype of stromal cells in the TME may also be affected by *BRCA1* mutation in tumor cells (225). Moreover, the BRCA1 loss mutation, like oncogene activation (RAS, NF-κB, TGF-β), in cancer cells will drive the initiation of metabolic symbiosis phenotype between tumor cells and fibroblasts in both primary and metastatic cancers (226).

#### PGC-1α

PGC-1α is a transcriptional co-activator that actively participates in gene regulation of energy metabolism. Elevated expression of PGC-1α in breast cancer is well associated with the formation of distant metastases. Notably, breast cancer cells with higher levels of PGC-1α may preferentially metastasize to some specific tissues, such as lung and bone (139). Silencing of PGC-1α appears to suspend cancer cell invasive potential and attenuate metastasis (137). The invasive cancer cells particularly do favor mitochondrial respiration with augmented production of ATP. As such, the circulating and metastatic cancer cells upregulate the PGC-1α to facilitate oxygen consumption rate oxidative phosphorylation, and mitochondrial biogenesis to uphold metastasis (137).

#### RB1

RB1* *is a tumor suppressor that is commonly disrupted in many human tumors, including breast cancer (227). *RB1 *deficiency is connected with cancer invasion and metastasis (228, 229). It is evaluated that *RB1* and *TP53* are lost together in 28–40% of human TNBCs, and *RB/P53*-double mutant mouse breast tumor cells exhibit more mesenchymal phenotypes than only *P53*-deficient cells (230, 231). *RB1* loss links to increased mitochondrial OXPHOS, which links to enhanced anabolic metabolism and augmented cancer cell stemness and metastatic spread (232). Additionally, *RB1 *deficiency is able to enhance tumor metastasis by increasing OXPHOS to generate more ATP fueling for tumor invasion and cooperating oncogenic alterations to uphold EMT and metastasis (232).

#### LKB1-AMPK Signaling

AMPK is a universally expressed metabolic sensor, which can be phosphorylated and activated under some stress conditions, such as energy deprivation. Phosphorylated AMPK activates multiple downstream elements to regulate adaptive changes and maintain metabolic homeostasis, including glucose, lipid or protein metabolism. Recently, the latent roles of AMPK signaling in tumorigenesis and progression have been gradually revealed (233). Activated AMPK signaling regulates protein and lipid synthesis by inhibiting mTORC1 through activation of tuberous sclerosis complex 2 (TSC2) and phosphorylation of raptor (234–237). The chief activator of AMPK is the serine-threonine tumor suppressor kinase LKB1, which contributes to phosphorylation of AMPK to activate energy sensors (235, 238). As long as LKB1-AMPK signaling is activated, the regulation of the metabolic branch of mTOR signaling cannot be impaired in spite of the abnormal of PI3K/AKT or receptor tyrosine kinase signaling (237). LKB1 inactivation has recently been reported to drive tumor progression by cooperating with certain activating oncogene mutations in various models of cancer (239–242). Lysine demethylase 5B (also known as KDM5B) is upregulated in breast tumors and play an important role in lipid metabolic reprogramming (243). A recent study clearly demonstrated the knockdown of KDM5B reversed the EMT process to inhibit breast tumor cell migration by activating AMPK signaling-mediated lipid metabolism (244).

### Extrinsic Factors: Interaction Between Metabolic Pathways/Fluxes and Breast Cancer Metastasis Induced by Hypoxia, Oxidative Stress, Acidosis, and Tumor Microenvironment

#### Hypoxia

Hypoxia represents an important characteristic in the TME arising as a mismatch between cellular oxygen consumption and supply (245). About 25%–40% of invasive breast tumors display hypoxic situations (246). Hypoxia is able to regulate glycolysis, glycogen synthesis, lipid metabolism and oxidative phosphorylation, thus playing a vital role in tumor cell survival and growth during all stages of metastasis (247).

Hypoxia-inducible factors, including HIF-1α and HIF-2α, are main regulators in adaptation to hypoxia and nutrient deprivation during tumor progression (141). The activated HIFs is able to induce the expression of various gene products, such as glycolysis- and EMT program-associated molecules (CXCR4, SNAIL and TWIST), the induced pluripotency-associated transcription factors (OCT-3/4, NANOG, and SOX2), angiogenic factors (VEGF) and microRNAs, which are vital to self-renewal, survival, invasion, metastasis, angiogenesis, metabolic reprogram, and treatment resistance of cancer cells. Furthermore, elevated HIF-1α level is a predictive marker of early relapse and metastasis, and correlated with bad clinical outcome in human breast cancer (248–250). Inhibition of HIF-1 activity has a significant inhibitory effect on primary tumor proliferation and metastasis to lymph nodes and lungs in mice by orthotopic transplantation of TNBC (251, 252). Notably, HIF-1 mediates adaptive metabolic responses to hypoxia by enhancing glycolytic pathway, serine synthesis and one-carbon metabolism to promote mitochondrial antioxidant production (NADPH and GSH), and inhibiting the TCA cycle so as to diminish mitochondrial ROS production (247). HIF-1α has recently been shown to increase the expression levels of pro-collagen prolyl (P4HA1 and P4HA2) and lysyl (PLOD1 and PLOD2) hydroxylases in both tumor and stromal cells, thereby enhancing cancer cell alignment along collagen fibers, thereby promoting invasion and metastasis to lymph nodes and lungs (253–255). Hypoxia raises the proportion of BCSCs in a HIF-1α–dependent manner (256, 257), which will contribute to cancer metastasis. A recent study demonstrated that HIF-1α appeared to dynamically regulate glucose metabolism based on oxygen availability to prevent the risk of continuous incremental ROS production to keep redox homeostasis. This HIF-1α-induced effect is vital for induction of the BCSC phenotype in breast cancer when in response to hypoxia or cytotoxic chemotherapy (33). PDK1, a HIF-1α target that antagonizes the function of PDH, a main rate-limiting enzyme for pyruvate converting to acetyl-coA and entering the TCA cycle, has been reported to be a critical regulator of breast cancer metabolism and metastasis (138). Liver metastatic breast cancer cells are recognized to depend on the HIF-1/PDK1 axis for their metabolic reprogramming to accelerate their efficient colonization and proliferation in the liver (138). Some metabolic enzymes, such as succinate dehydrogenase (SDH), fumarate hydratase (FH), IDH and pyruvate kinase 2 (PKM2) are likely to activate HIF-1 pathway by stabilizing HIF-1α, therefore enhancing cancer metastasis (258).

#### Reactive Oxygen Species and Antioxidants

Tumor cells can only survive within a narrow window of ROS levels. Inhibition of ROS clearance is a therapeutic approach  (259), and on the contrary, prohibition of ROS enhances tumor metastasis (260). Among the detachment from ECM during the procedure of metastasis, cancer cells can undergo alterations in metabolic pathways harmful to survival, such as moderated glucose uptake, PPP flux, and ATP levels while promoting the producing of ROS (261). Antioxidant enzymes support survival of breast tumor cells deprived of ECM, implying that eliminating antioxidant enzyme activity in ECM-detached tumor cells may be an efficacious strategy to stop metastatic spreading (262). Additionally, the untransformed breast epithelial cells upregulate PDK4 to inhibit PDH and attenuate the flux of glycolytic carbon into mitochondrial oxidation, consequently suppressing anoikis (the absence of the home environment) upon detachment from ECM (261). By stimulating PDH in cancer cells to normalize glucose metabolism, it can restore their sensitivity to anoikis and weaken their metastatic potential, suggesting that PDKs are potential targets for anti-metastasis therapy (261). Another way to counter increased ROS production in breast cancer cells is to induce the expression of catalases, such as manganese superoxide dismutase (MnSOD). The expression of MnSOD is elevated in metastatic breast cancer, and its overexpression is correlated with histologic tumor grades (263). Isaac et al. has suggested that combined inhibition of endogenous antioxidant GSH and thioredoxin antioxidant pathways can produce a synergistic anti-cancer effect both *in vitro* and *in vivo* (264).

### Extracellular Acidification

Lactate, the final product of glycolysis, is released from cells together with H^+^ ions by means of monocarboxylate transporters and hydrogen ion pumps, and the excess carbon dioxide produced in the process of mitochondrial metabolism diffuses into the extracellular space and is then converted into H + and HCO3- by carbonic anhydrase (265). In situations of metabolic stress, such as nutrient deprivation and hypoxia, these reactions are strengthened, leading to extracellular acidification and enhancing the proteolytic activity of MMPs. Afterwards, the ECM is remodeled, which facilitates tumor invasion (265, 266). It has been reported that extracellular lactate also increase tumor invasion and metastasis by facilitating the fibroblast expression of hyaluronan and CD44 (267). Besides, increased extracellular lactate induces tumor-associated stromal cells to secrete VEGF, thus reinforcing angiogenesis (268). The augment in extracellular lactate has also been reported to provide an immune-conducive environment for tumor cells by reducing the activation and function of dendrites and T cells (269, 270).

#### Cancer-Associated Fibroblasts

Cancer-asscociated fibroblasts (CAFs), the paramount stromal cells in breast tumor microenvironment, contribute to tumor progression through many mechanisms, such as releasing of assorted secretory proteins (e.g. TGF-β, IGF, and IL6), direct interplaying with tumor cells, regulating immune-response, ECM remodeling, and inducing cancer metabolic reprogramming (271). Breast cancer cells MCF-7 exhibited increased aerobic glycolysis when co-cultured with adjacent fibroblasts. Mechanically, the lactate produced by the CAFs can be used by cancer cells, thus enhancing aerobic glycolysis, which is called the “reverse Warburg effect” (28, 272). Similarly, metabolomic analysis showed that CAFs also produce glutamine and other metabolites that can be utilized by tumor cells (273). Subsequent researches demonstrated that co-culture with MCF-7 and CAFs resulted in promoted glutamine catabolism and inhibited glutamine synthesis in cancer cells, thereby promoting cancer cell growth and progression (274).

#### Cancer-Associated Adipocytes

The “cancer-associated adipocytes (CAAs)” are generated by the transformation of tumor adjacent adipocytes (275). It has been reported that tumor-surrounding adipocytes exhibit an distinct phenotype comparing to normal adipocytes, which is characterized by upregulated beige/brown adipose markers and increased catabolism and the release of metabolites, including lactate, free fatty acids, pyruvate, and ketone bodies. Importantly, the tumor-adipocyte interaction can reprogram energy metabolism and foster tumor progression (276).

Accumulation of lipids is found in breast tumor cells when co-cultured with adipocytes (277). Tumor cells can switch from glycolysis to lipid-dependent energy production and also store excess lipids, which provides energy to support their expansion and metastasis (278). The ketone bodies produced and released by glycolytic fat cells are the ideal fuel for ATP production and they can be burned more efficiently than other mitochondrial substrates, even during hypoxia, potentially allowing the tumor grow when without adequate blood supply (279). It is worth noting that the co-existence of adipocytes and cancer cells enhances both ketogenesis in adipocytes and ketolytic activity in breast cancer cells (276). In addition, β-hydroxybutyrate secreted from adipocytes is able to induce several tumor-promoting genes in breast cells, and facilitate breast tumor cells malignant growth *in vitro* (280). Moreover, elevated ketone-specific gene expression is related with worse outcomes in breast cancer patients (281).

#### Immune cells in Tumor Microenvironment

The local immune surveillance environment is increasingly recognized as a significant factor inhibiting tumor metastasis. Apart from fundamental competition for nutrients required by cancer cells and immune cells in TME, metabolic pathways change in tumor cells may influence tumor-infiltrating immune cells, and different immune cell subgroups in TME have specific metabolic characteristics (282). The limitation of glucose and amino acids within the TME can significantly affect the T cell response and the determinants of metabolic dysfunction and associated T cell exhaustion within the TME are also being explored. Studies have shown that cancer itself can cause effector T (Teff) cell metabolism disorders, and there is a negative correlation between the degree of glycolytic activity of cancer cells and the antitumor function of infiltrating T cells (282). A study has confirmed that the expression of glycolysis-related genes in tumor samples from patients with melanoma and non-small-cell lung cancer is negatively correlated with T cell infiltration, and that tumor glycolysis is related to the efficacy of adoptive T cell therapy (ACT), suggesting that the glycolytic pathway may be a candidate target for combined therapeutic intervention (283). Inhibition of cholesterol esterification in T cells by genetic ablation or pharmacological inhibition of ACAT1 (a key cholesterol esterase) can lead to potentiated effector function and enhanced proliferation of CD8(+) T cells by increasing plasma membrane cholesterol levels, which causes enhanced T-cell receptor clustering and signaling as well as more efficient formation of the immunological synapse, thereby controlling the growth and metastasis of mouse melanoma (284). However, such studies are still lacking in breast cancer. Tumor-derived myeloid-derived suppressor cells (MDSCs) are critical tumor immunosuppression components. Glycolysis restriction limited the development of MDSCs by inhibiting tumor expression of granulocyte colony-stimulating factor (G-CSF) and granulocyte macrophage colony-stimulating factor (GM-CSF), therefore enhanced T cell immunity, reduced tumor growth and metastasis, and prolonged survival in two TNBC mouse models (285). Interestingly, hypoxia through HIF-1α significantly changes the function of MDSC in TME and shifts its differentiation direction to tumor-associated macrophages (TAMs) (286). TAMs are well-known parts of breast cancer microenvironment and most TAMs within TME are closely related to the M2-like phenotype, which participate in almost all metastatic processes, including local invasion, blood vessel intravasation, extravasation at distant sites and metastatic cell growth (287, 288).The hypoxic areas in tumors are related to the accumulation of macrophages, which assist tumor progression by producing angiogenic factors, mitogenic factors and cytokines related to tumor metastasis (289–291). In addition, hypoxia can promote the differentiation and functional capabilities of immunosuppressive macrophages (292). Blockade of Eotaxin/Oncostatin M not only prevented hypoxic breast tumor cells from recruiting and polarizing macrophages towards the M2-like phenotype and hindered cancer progression in 4T1 breast cancer model but also improved the efficacy of antiangiogenic Bevacizumab, suggesting these two cytokines as novel targets for devising effective anticancer therapy (293). Lactic acid production by tumor cells, as a byproduct of aerobic or anaerobic glycolysis, has also been shown to play a vital role in the M2-like polarization of TAMs, which is mediated by HIF- 1α (294).

The metabolites produced by cancer cells may hinder the antitumor immune response by affecting different tumor infiltrating immune cells (295). Cholesterol metabolites, oxysterols, which act as endogenous regulators of lipid metabolism through the interaction with the nuclear Liver X Receptors-(LXR)α and LXRβ, aid tumor progression by inhibiting antitumor immune responses, and by recruiting proangiogenic and immunosuppressive neutrophils. A recent study showed that in the 4T1 breast cancer model the enzymatic depletion of oxysterols in primary tumors decreases the formation of lung metastases by regulating the levels of immune cells infiltrating the metastatic TME, and tumor-associated neutrophils are the main driving force of local immunosuppression (296). Another work also proved that by recruiting immunosuppressive neutrophils in the metastatic niche, oxysterol 27-HC played a role in promoting metastasis in breast cancer models (297).

## Drugs Targeting Metabolism in Metastatic Breast Cancer

Breast cancer patients who have not yet found metastasis are at high risk of metastasis, and those metastatic breast cancers are not curable due to lack of effective treatments. Early intervention in the early stage of distant metastasis, during the period of colonization and growth will be more beneficial to the survival of the patient. There are many promising drugs targeting altered metabolism pathways undergoing disparate stages of preclinical studies and clinical trials (**Table 2**). However, there is currently no clear conclusion of the clinical benefit of metabolic interfering drugs in the treatment of breast cancer. A Phase II clinical trial involving 164 patients was recently showed that in patients with HER2-metastatic breast cancer, addition of indoximod, the** **Indoleamine 2,3-dioxygenase 1 (IDO1) pathway inhibitor, to taxane did not improve PFS compared with taxane alone (300). The use of glucose metabolic inhibitors such as 2-deoxy-D-glucose (2-DG) and metformin in combination with chemotherapy has shown encouraging results in combating chemotherapy resistance (301). One patient with medullary breast cancer metastatic to lung and lymph nodes underwent extensive pretreatment (8 previous systemic treatment options) was reported to have a confirmed partial response (PR) with a duration of 65 days when treated with 45 mg/kg 2-DG every other week (298). Dichloroacetate (DCA) can enhance metformin-induced oxidative damage with simultaneous reduce of metformin promoted lactate production through PDK1 inhibition, suggesting the innovative combinations, such as metformin and DCA, will be promising in expanding breast cancer therapies (302). Nevertheless, due to the limited sample and the lack of evidence for benefit, further researches are needed.

**Table 2:** Current metabolic interventions in metastatic breast cancer.

Targeting	Drugs	Phase	Populations	Clinical Trials	Study Date	Results
Glucose metabolism						
Hexokinase	2-deoxy-d-glucose (2DG) (alone and combined with docetaxel)	Phase I	Locally advanced or metastatic solid tumors including breast cancer	NCT00096707	2004.02–2008.07	Feasible but need further evidence (298)
Pyruvate dehydrogenase kinase(PDK)	Dichloroacetate (DCA)	Phase II	Previously Treated metastatic breast cancer or non-small-cell lung cancer.	NCT01029925	2009.12–2011.11	Suspended
Complex I	Metformin	Phase I/II/III	All breast cancer	Multiple clinical trials		Feasible but need further evidence (299)
Lipid metabolism						
FASN	TVB-2640 (combined with paclitaxel and trastuzumab)	Phase II	HER2+ metastatic breast cancer resistant to trastuzumab and taxane-based therapy	NCT03179904	2017.08-	Unpublished
Amino acid metabolism						
Glutaminase	CB-839	Phase I	Advanced solid tumors including triple-negative breast cancer	NCT02071862	2014.02–2019.03	Unpublished
	CB-839 (combined with paclitaxel)	Phase II	Locally-advanced or metastatic triple-negative breast cancer	NCT03057600	2017.05–2019.11	Unpublished
Indoleamine 2,3 dioxygenase (IDO1)	Indoximod (combined standard of care therapy (docetaxel or paclitaxel))	Phase II	HER2- metastatic breast cancer	NCT01792050	2013.02–2017.07	Cannot improve PFS (300)
	Indoximod	Phase II	metastatic invasive breast cancer that is positive for p53 staining by IHC (>= 5%)	NCT01042535	2009.12–2018.02	Unpublished
Arginine deiminase (ADI)	ADI-PEG20	Phase I	HER2- metastatic breast cancer or advanced solid tumor	NCT01948843	2014.04–2016.04	Unpublished

Metabolic inhibitors combined with checkpoint inhibitors holds promise to enhance the efficacy of immunotherapy and the relationship of tumor-intrinsic metabolism and successful immunotherapy is being explored. Tumor-imposed metabolic restrictions can mediate T cell hyporesponsiveness during cancer. Checkpoint blockade antibodies against CTLA-4, PD-1, and PD-L1, can restore glucose in tumor microenvironment, permitting T cell glycolysis and IFN-γ production, and blocking PD-L1 directly on tumors dampens glycolysis by inhibiting mTOR activity and decreasing expression of glycolysis enzymes (270). Because breast cancer immunotherapy is in the ascendant, understanding the metabolic dependence between infiltrating immune cells and cancer is an important direction for future research.

For ER-positive breast cancer patients, endocrine therapy is very beneficial, but some patients will develop endocrine therapy resistance. Whether endocrine therapy combined with metabolic therapy will achieve better results still needs a lot of preclinical studies and clinical trials to verify. It has been reported that trastuzumab resistant cells exhibit enhanced glycolysis phenotype, and glycolytic restraint is able to sensitize trastuzumab resistant HER2+ breast cancers to trastuzumab treatment (303). The TN/basal-like breast cancer lacks the therapeutic targets, and chemotherapy is currently the main treatment strategies. Based on the TNBC unique metabolic phenotype, there are many existing researches focus on the metabolic interference in chemotherapy resistance models and spontaneously metastatic preclinical models (304, 305). What is more, the metabolic characteristics of tumor cells and their microenvironment in different metastatic sites are different, therefore, the corresponding targeting treatment plans can also be considered in the future (306).

Although anti-cancer therapy targeting metabolism has achieved some gratifying results, it is still currently believed that this field has the following shortcomings for possible future breakthroughs: 1) The side effects of such drugs limit their clinical effects as the optimal dose window is hard to be determined; 2) Due to the extremely complex signaling pathways in the regulation of normal cellular biology, inhibition of a specific signaling pathway will definitely have feedback activation or upregulation of other alternative signaling pathways, therefore causing ultimately treatment failure; 3) To specifically target related mutations involved in metabolic pathways is challenging. 4) Accurate screening of the beneficiaries is the key to improve the drug effect, and is an urgent problem to be solved in the future.

## Conclusions

Metabolic programming supports several steps of successful metastasis in breast cancer. Breast cancer cells exhibit different metabolic phenotypes in different metastasis sites. Both intrinsic factors, properties arising in the malignant cells, such as *MYC* amplification, *PIK3CA*, and *TP53* mutations, and extrinsic factors, metabolic stresses imposed by the microenvironment, such as hypoxia, oxidative stress, acidosis, contribute to different metabolic programming phenotypes in metastatic breast cancer. More importantly, interfering with tumor metabolism to control tumor progression is a very promising approach in cancer treatment, although it is full of challenges. More researches are required to further discover the related genes and molecular mechanisms involved in metabolism reprograming during cancer progression, so that they can be used for targeting therapy in clinical practice in the future. We also look forward to further advances in approaches to judge and quantify metabolic phenotypes in human breast cancers *in vivo*, including metabolomics, metabolic imaging and isotope tracing studies, so that clinical oncologists will develop treatment strategies by matching the treatment to the patient-specific tumor metabolic characteristics.

## Author Contributions

LW wrote the first draft of the manuscript. All authors contributed to the article and approved the submitted version.

## Funding

This work was supported by the Zhejiang Provincial Natural Science Foundation of China (grant no. Y19H160283).

## Conflict of Interest

The authors declare that the research was conducted in the absence of any commercial or financial relationships that could be construed as a potential conflict of interest.
